# Changes in error rates in the Australian key incident monitoring and management system program

**DOI:** 10.11613/BM.2020.020704

**Published:** 2020-04-15

**Authors:** Stephanie Gay, Tony Badrick

**Affiliations:** Royal College of Pathologists of Australasia Quality Assurance Programs (RCPAQAP), Key Incident Monitoring and Management System program (KIMMS), Sydney, Australia

**Keywords:** preanalytical errors, postanalytical errors, preanalytical risk, postanalytical risk

## Abstract

**Introduction:**

The Key incident monitoring and management system program (KIMMS) program collects data for 19 quality indicators (QIs) from Australian medical laboratories. This paper aims to review the data submitted to see whether the number of errors with a higher risk priority number (RPN) have been reduced in preference to those with a lower RPN, and to calculate the cost of these errors.

**Materials and methods:**

Data for QIs from 60 laboratories collected through the KIMMS program from 2015 until 2018 were retrospectively reviewed. The results for each QI were averaged for the four-year average and coefficient of variation. To review the changes in QI frequency, the yearly averages for 2015 and 2018 were compared. By dividing the total RPN by 4 and multiplying that number by the cost of recollection of 30 AUD, it was possible to assign the risk cost of these errors.

**Results:**

The analysis showed a drop in the overall frequency of incidents (6.5%), but a larger drop in risk (9.4%) over the period investigated. Recollections *per* year in Australia cost the healthcare industry 27 million AUD. If the RPN data is used, this cost increases to 66 million AUD *per* year.

**Conclusions:**

Errors with a higher RPN have fallen more than those with lower RPN. The data shows that the errors associated with phlebotomy are the ones that have most improved. Further improvements require a better understanding of the root cause of the errors and to achieve this, work is required in the collection of the data to establish best-practice guidelines.

## Introduction

Pre- and postanalytical errors are now widely accepted as being the most common source of error in laboratory medicine (*1*, *2*). In response to this awareness, many laboratories track these errors, and use external quality assurance programs such as Key incident monitoring and management system (KIMMS) or the International Federation of Clinical Chemistry – Model of quality indicators (IFCC-MQI) (*3*, *4*). Not all errors present the same level of risk for the patient, for example, a tube not collected is not as significant a risk to a patient as an incorrectly labelled tube. Similarly, a haemolysed specimen identified and recollected will have less impact on a patient’s clinical outcome than a haemolysed specimen analysed with results reported. Therefore, there needs to be a structured way to convert the frequency of error into the risk of the outcome.

The KIMMS program monitors errors that occur in the pre- and postanalytical testing phases that could lead to patient harm (outcome) (*3*). Attempts to monitor actual outcomes has not proved easy. The KIMMS program has approached this problem by using the failure mode and effects analysis (FMEA) technique to convert the frequency of incidents into a measure of potential patient harm (*5*).

Failure mode and effects analysis was developed in the 1950s as a highly structured, systematic technique for failure analysis in engineering. A risk priority number (RPN) is derived from the product of the frequency of failures detected, the relative ability to detect failures, and how serious are their consequences (harm). This approach allows resources to be deployed most effectively by prioritising the most significant errors *i.e.* those with the highest RPN. Failure mode and effects analysis is now a recognized process analysis tool and widely used in industry, government and medical process improvement methods. It was first used in the health industry in the 1990s and provided an indirect connection between error (or incident), causes and harm (or poor patient outcomes) (*5*, *6*). This paper aims to review the data submitted to the KIMMS program to see whether the number of errors with a higher RPN have been reduced in preference to those with a lower RPN, and to calculate the cost of these errors.

## Materials and methods

Sixty Australian medical laboratories, either as an individual laboratory or a group of laboratories using the same laboratory information system (LIS) (known as participants), code episodes that have errors associated with them; the code used indicates the nature of the error. Each type of error is known as a quality indicator (QI). From 2015 until 2018, KIMMS collected data for 19 Quality Indicators (QIs). Participants extract the frequency of each QI from their LIS and enter this information manually via the Royal College of Pathologists of Australasia Quality Assurance Programs (RCPAQAP) data entry portal. The information is uploaded into the KIMMS database. A report is produced using the RCPAQAP QDS program. The QDS program was produced in house in 1995, initially for chemical pathology programs, and extended for use by the KIMMS program in 2011. The report includes the frequency of each QI as submitted by the participant and the RPN for each QI.

To calculate the RPN, the KIMMS Advisory Committee assigned a numerical value to the likelihood of finding the error (Table 1) and the possible harm to the patient (Table 2). The values are empirical but represent an exponential scale of possible harm and error detection. By multiplying the frequency of an error by the likelihood of detection and by the possible harm, the RPN for each type of error is calculated. The KIMMS program refers to the multiplication of harm by detection as the “risk factor.”

**Table 1:** Likelihood of detecting an error

**Detection (value)**	**Definition**
Almost certain (1)	This error is impossible to miss.
Likely (4)	If processes are followed, this error will be found.
Unlikely (7)	This error will only be found by luck.
Rare (10)	This error will only be found if another error occurs to bring it to light.
The Key incident monitoring and management system program (KIMMS) Advisory Committee assigned a name and a numerical value to the likelihood of finding the error (detection and value). The values are empirical but represent an exponential scale of error detection. They are used in the calculation of risk priority number (RPN).	

**Table 2:** Likelihood of harm to the patient

**Harm (value)**	**Definition**
Not significant (1)	No long term effects to the patient are expected.
Recollection (4)	The patient will suffer the risk associated with any collection.
Delay (7)	The patient is likely to suffer a delay in diagnosis.
Not diagnosed (10)	The patient is likely not to be diagnosed correctly.
The Key incident monitoring and management system program (KIMMS) Advisory Committee assigned term and a numerical value to the amount of harm to the patient *e.g.* the likely long-term outcome (harm and value). The values are empirical but represent an exponential scale of possible harm. They are used in the calculation of risk priority number (RPN).	

To ensure that the data presented is representative of Australian laboratories, the statistics supplied by Medicare (the funder of private and community pathology in Australia, including private hospitals) and an estimate of the amount of pathology performed by the public system (public hospitals) by Pilbeam *et al.* were combined to calculate the percentage of episodes collected by KIMMS (*7*, *8*). Medicare defines an episode as “pathology services requested by a practitioner in respect of one individual on the same day” (*7*).

The data is reported to KIMMS each quarter. The period covered in this report is from 2015 to 2018, during which time the QIs did not change. The RCPAQAP QDS software can extract the data for each participant by QI. The results for each QI were reviewed, and any outliers removed. Any single result that was more than ten times different from the previous and following quarters result was considered an outlier.

The results for each QI were averaged for each quarter, and these results were averaged for the four-year average and coefficient of variation (CV). To review the changes in QI frequency, the yearly average for 2015 was compared to the yearly average for 2018. All calculations were performed using Microsoft excel 2016.

To calculate the cost of these errors, the cost of performing a recollection was required. Green estimated the average cost of a preanalytical error to be 208 USD in North America and 204 USD in Europe. This cost is equivalent to 295 AUD, of which, according to Green, between 4 and 10 percent can be attributed to recollection and laboratory costs, *i.e.* 11.80 AUD to 29.50 AUD (*9*). In Australia, up to 60% of pathology collections are not performed in hospital and the cost of contacting the patient and organising a recollection are higher than in a hospital situation. Thus, for this study, we estimated the cost of recollection to be 30 AUD. The frequency of errors multiplied by the cost of a recollection is the most straightforward method of assessment of cost but does not consider the different risk factors of each error. The second method of assessment is to relate the RPN back to the risk factor for a recollection, which has been set at 4. By dividing the total RPN by 4 and multiplying that number by 30 AUD, it was possible to assign the risk cost of these errors.

## Results

Medicare reports 38 million episodes were funded in 2017/2018 and Pilbeam *et al.* suggest up to 40% of the total pathology market is in the public domain (*8*, *10*). 100% is equivalent to 256 million episodes in 4 years. The KIMMS program had 166 million episodes or 65% reported in the same time frame. The 19 QIs reviewed are listed in Table 3, along with the average result *per* 1000 episodes for 4 years. The total number of results (N) and the number of outliers (O) are also recorded. The last 5 QI’s shown in Table 3 have not been included in the calculations since the results for each year showed inconsistencies, as reflected in the higher CVs (see Discussion).

**Table 3:** Quality indicators used in the Key incident monitoring and management system program

**QI included in calculations**	**4 year average *per* 1000 episodes**	**CV (%)**	**N**	**O**
Haemolysed samples	7.7	7	820	12
Sample not collected	3.17	6	814	3
Clotted samples	1.93	6	825	4
Insufficient sample	1.65	9	820	4
Discrepancy of ID	1.00	19	818	10
Incorrect sample type	1.09	6	813	4
Incorrect fill of samples	0.89	21	811	1
Transfusion sample ID	0.83	11	763	4
Unlabelled	0.84	10	823	0
Incorrect storage or transport	0.55	12	808	3
Insufficient Identifiers	0.39	15	781	0
Sample contaminated	0.23	23	773	0
Report sent to wrong Dr	0.14	32	651	0
WSIT	0.10	14	608	2
**QI excluded from calculations**				
Precious samples	0.15	25	738	4
Within laboratory ID error	0.11	57	771	1
Laboratory accident	0.25	32	805	1
Report retracted (amended)	0.31	32	723	3
Registration incidents	1.3	28	803	1
QI – quality indicators. CV – coefficient of variation. O – outliers removed for each QI. ID - patient identifiers. Dr – requesting medical practitioner. WSIT- wrong sample in tube (tube and paperwork labelled with matching, but incorrect patient identifiers). The 4-year average and CV were calculated from the average result for each quarter for each QI for the years 2015 to 2018 inclusive. Prior to this calculation, results considered to be outliers where removed. These are results for individual participants that differed by more than ten times other results for them. The number of results received (N) shows the total number or results received in the 4-year period for each QI (minus the outliers) out of a total possible of 832.				

The CVs for the other fourteen QIs vary from 6 - 32%. It is not possible to know why some vary more than others. However, there is a tendency for those QIs with fewer results recorded to have higher CVs.

The yearly breakdown for the 14 QIs and the RPN for each of these is shown in Table 4. These results show that the top five errors for frequency are not the same as the top five errors for RPN. Haemolysis is the highest risk for both. Sample not collected, sample clotted, Insufficient sample, and discrepancy in identification (ID) are the next top four errors for frequency, while discrepancy in ID, incorrect fill, sample not collected and incorrect storage and transport are the next top four errors for RPN.

**Table 4:** Frequency and risk priority number *per* 1000 episodes by year for each quality indicator

**QI**	**Frequency *per* 1000 episodes**				**RPN *per* 1000 episodes**			
**Year**	**2015**	**2016**	**2017**	**2018**	**2015**	**2016**	**2017**	**2018**
Haemolysed samples	7.9	7.9	7.7	7.4	126	126	123	118
Sample not collected	3.32	3.09	3.06	3.2	13.3	12.4	12.2	12.8
Clotted samples	2.05	1.83	1.85	1.96	8.2	7.3	7.4	7.8
Insufficient sample	1.58	1.72	1.55	1.71	6.3	6.9	6.2	6.8
Discrepancy of ID	1.19	1.11	0.9	0.76	19.1	17.8	14.4	12.2
Incorrect sample type	1.09	1.07	1.08	1.11	4.4	4.3	4.3	4.4
Incorrect fill of samples	1.12	0.91	0.74	0.75	17.9	14.6	11.8	12
Transfusion sample ID	0.84	0.79	0.77	0.89	3.4	3.2	3.1	3.6
Unlabelled	0.85	0.74	0.89	0.91	3.4	2.96	3.6	3.6
Incorrect storage or transport	0.59	0.55	0.53	0.54	9.4	8.8	8.5	8.6
Insufficient Identifiers	0.33	0.36	0.48	0.42	5.3	5.8	7.7	6.7
Sample contaminated	0.26	0.25	0.17	0.22	4.1	4	2.7	3.5
Report sent to wrong Dr	0.16	0.11	0.17	0.14	2.56	1.76	2.72	2.24
WSIT	0.09	0.09	0.10	0.09	9.1	9.2	10.5	9.2
Total	21.4	20.5	20.0	20.0	232	225	218	211
QI – quality indicators. RPN – risk priority number. ID - patient identifiers. Dr – requesting medical practitioner. WSIT- wrong sample in tube (tube and paperwork labelled with matching, but incorrect patient identifiers). The frequency is the actual number of errors reported by Key incident monitoring and management System program participants and the RPN is calculated by multiplying frequency, likelihood of detection and possible harm.								

In 2015, there were 696,409 errors reported from 32,447,679 episodes, equal to a rate of 21.4 errors *per* 1000 episodes. By 2018, this had grown to 910,438 errors from 45,433,742 episodes, which is a rate of 20.0 incidents *per* 1000 episodes, an overall reduction of 6.5%. Over the same time interval, the RPN fell from 233 *per* 1000 episodes to 211 *per* 1000 episodes, which is a 9.4% fall. A breakdown of the changes for each QI is shown in Table 5. This shows that there is variation in the amount of change for each.

**Table 5:** Changes in risk priority number between 2015 and 2018 for the Key incident monitoring and management system program quality indicators

**QI**	**Reduction of the overall change in RPN (%)**	**QI**	**Increase of the overall change in RPN (%)**
Haemolysed samples	31.0	Insufficient identifiers	5.4
Discrepancy of ID	27.0	Insufficient sample	1.9
Incorrect fill of samples	23.0	Unlabelled	0.8
Storage or transport of sample	3.1	Transfusion ID issues	0.8
Sample contaminated	2.3	WSIT	0.4
Sample not collected	1.9		
Clotted samples	1.6	**QI**	**No overall change in RPN (%)**
Report sent to wrong Dr	1.2	Incorrect sample type	0
QI – quality indicators. RPN - risk priority number. ID - patient identifiers. Dr – requesting medical practitioner. WSIT- wrong sample in tube (tube and paperwork labelled with matching, but incorrect patient identifiers). The overall reduction of 9.5% in the RPN from 2015 compared to 2018 is not evenly spread between the QIs.			

## Discussion

The importance of setting and monitoring QIs has been emphasized but as yet there have been few publications about the impact of this approach on reducing error rates although, generally speaking, monitoring does lead to improvement (*11*, *12*). The preanalytical errors with the greatest frequency were haemolysis, sample not collected, sample clotted, incorrect sample type, insufficient sample, and insufficient identification as previously identified by Plebani, Sciacovelli, Aita *et al* (*13*, *14*). The collection of QIs can be problematic for laboratories as often LIS systems are not set up to adequately identify different error types, for example to be able differentiate between an amended result due to it being incorrect or additional information being added to the report. An incorrect result will be recorded in the Quality system rather than the LIS. A single error in the laboratory (*e.g.,* transcription error, pipetting error, equipment malfunction) may result in more than one report being amended. All of these reasons can lead to inconsistent results for a participant.

There are also variations in the way that laboratories classify errors despite attempts to standardise nomenclature (*3*, *11*). For example, precious sample ID errors and registration errors are interpreted differently by different participants. Exactly what should be included in precious samples needs to be better defined (should it include microbiology samples that can’t be recollected due to antibiotic commencement, should paediatric samples be included). Registration errors include patient identification information wrongly entered, test codes incorrect or missed and doctor’s codes incorrect or missed; but does it also include lack of clinical information, missed times of collection and drug dosage information? In 2019, the precious sample QI was removed due to these inconsistencies. In 2020, registration errors will be further broken down to reflect the nature of the error as will amended result information.

There are different ways to try and evaluate error including measuring frequency, sigma metrics, cost (*9*, *13*-*15*). The KIMMS program uses a FMEA process to prioritise error based on harm (*3*). Applying the risk matrix and calculating the RPN for each incident may have helped to focus action on the areas of greatest risk. The higher drop in risk (9.4%) compared to frequency (6.5%) indicates that the change in each QI is not equal and that overall, those QIs with a higher RPN fell more than those with lower RPN.

It is not possible for KIMMS to be able to answer the question “why the frequency and risk have dropped?” however, individual participants should know why their frequencies and risks have changed. A possible explanation is an improvement in phlebotomist competence (*e.g.* 6 out of 8 QIs that have improved are phlebotomy related), which has been driven by a greater focus on phlebotomist training (*8*). Similarly, reporting risk as well as frequency data has helped to raise the profile of the low frequency, high harm incidents such as “the wrong sample in tube” (WSIT).

Table 6 shows the difference in the costs of the average incidents *per* year calculated by both measures – frequency and RPN. These show a cost to the Australian Health Care industry of 27 million AUD based on frequency, or 66 million AUD if they use RPN to calculate. The savings of 6.5% is equivalent to 1.7 million AUD and 9.4% savings is equivalent to 6.2 million AUD. 66 million AUD equates to a cost of 72 AUD *per* error, which is one quarter the cost of 295 AUD suggested by Green (*9*). Further work in the area of the cost of error in Australia is required.

**Table 6:** Cost of pre- and postanalytical error: comparison of cost based on the number of errors and cost based on risk priority number

	**Cost using number of errors (frequency)**	**Cost using RPN (risk)**
Average episodes (2015 - 2018)	40,977,248	40,977,248
Averge incidents (2015 - 2018)	913,792	/
Average RPN (2015 - 1018)	/	8,810,108
Total cost 30 AUD/recollection	27,413,760	/
Total cost 30 AUD/4 RPN	/	66,075,810
Savings for 6.5% reduction in incidents, AUD	1,781,894	/
Savings for 9.4% reduction in risk, AUD	/	6,211,126
RPN - risk priority number. The cost of pre- and postanalytical errors can be calculated by multiplying the cost of a recollection (30 AUD) by the number of errors. This does not take into account that different errors can cause more harm to the patient than others. A second method of calculating the cost is to divide the total RPN *per* year by 4 (the RPN for a recollection) and multiplying that by the cost of a recollection. / - not applicable.		

Using RPN as a measure of risk is not without its complications. The KIMMS program measures “Key” incidents that occur throughout the total testing cycle. The RPN measures the outcomes of incidents (harm) *i.e.* recollection, delayed treatment or wrong/no diagnosis, a useful tool to prioritize resources. However, to reduce errors, the root causes of incidents needs to be understood. The KIMMS program does not address this question in depth.

As a benchmarking tool for risk, KIMMS, in its current form, is not useful. The KIMMS program assumes constant harm and the likelihood of detection. With the introduction of changes such as technology, training and processes, there may be an increase in the ability to detect incidents for an individual site, which would reduce the RPN for that QI. The KIMMS program will not reflect this improvement. A laboratory better able to detect an incident will show an increase in frequency and thus an increase in RPN. The KIMMS program is unable to differentiate the two, and thus, measurement of individual risk compared to other participants is not valid.

The value of the KIMMS data is as a monitoring tool in a specific site. Each laboratory needs to monitor their own risk for each QI over time. When a process change is made, the expectation of change in the KIMMS data should be recorded, *i.e.* is the expectation that there will be an increase or decrease in the frequency of the QI. To calculate the risk, the laboratory should alter the ease of identity rating and perform its own calculation. Any known changes should have been documented and any unexpected changes investigated thoroughly. It is an unexpected change that is most likely to lead to the biggest increase in risk to the organisation.

Technologies available to laboratories to help with detection of errors include the use of label producing software to label tubes and record what has been collected, temperature monitoring devices that can be used during transport, and instruments to measure the levels of haemolysis, lipemia and icterus (*16*-*18*).

While KIMMS data as it is can be used by individual organisations to monitor the key QIs, it needs to be improved to allow benchmarking between organisations. It is not feasible to investigate 914,000 errors. However, it is possible to collect data along with the incident that will allow investigation of the cause. RCPAQAP is implementing direct download of results to the KIMMS database, which will facilitate the collection of de-identified data. Filters can then be applied to the frequency of the QIs which can then be investigated by such things as laboratory demographics (complexity, state, location: remote, regional, metropolitan), sample type, test requested, source of the sample (hospital, community), ward type, phlebotomist employer and requestor (specialist, general practice, hospital clinician). Provided more than five participants match the search criteria, participants will be able to compare themselves to their peers. This will allow laboratories to investigate their situation. Similarly, participants in the KIMMS program should be able to add information regarding how errors are detected allowing for more accurate calculation of RPN.

In conclusion, the use of RPN data to calculate the cost of errors to the pathology sector may prove to be a better indicator of the true cost of errors. There is a need for an accurate cost of a recollection to be calculated, both in the hospital and community domain in Australia. The data shows that the errors associated with phlebotomy are the ones that have most improved. There is a bigger fall in in RPN than in frequency, indicating that the errors with a higher RPN have fallen more than those with lower RPN, however, the changes are not uniform. Further improvements require a better understanding of the root cause of the errors and to achieve this, work is required in the collection of the data to establish best-practice guidelines.
